# Supplementary material for: Extremely Low-Frequency Electromagnetic Stimulation (ELF-EMS) Improves Neurological Outcome and Reduces Microglial Reactivity in a Rodent Model of Global Transient Stroke
Source: Int J Mol Sci. 2023 Jul 5;24(13):11117. doi: 10.3390/ijms241311117 (PMC10342400; doi:10.3390/ijms241311117)
Supplement: Supplementary file 1 [file ijms-24-11117-s001.zip › ijms-2456697-supplementary.pdf]

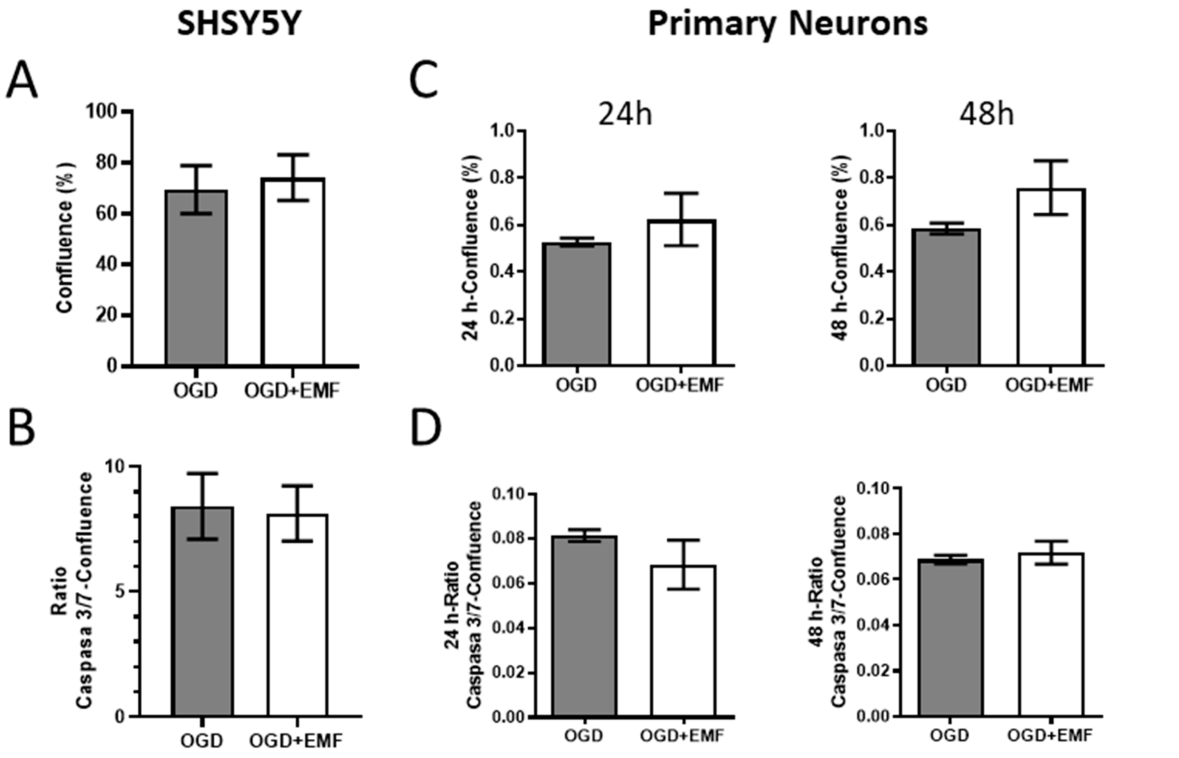

Supplementary Figure S1 ELF-EMS does not affect survival on neuron monocultures. *SH-SY5Y and primary neuron cells were submitted to 2 h OGD (1% O_2 and 5% CO_2, 30 min after the treated group was stimulated with 13.5 mT/ 60 Hz ELF-EMS for 20 min. Confluence and caspase 3/7 were measured 24 h and 48 h after OGD* ***(A)*** *Confluence of SH-SY5Y cell cultures 24 h after OGD* ***(B).*** *The ratio between % Caspase 3/7 and %Confluence 24 h after OGD in SH-SY5Y cells.* ***(C)*** *The confluence of primary neurons cultures 24 h (left) and 48 h (right) after OGD* ***(D).*** *The ratio between % Caspase 3/7 and %Confluence 24 h (left) and 48 h (right) after OGD in primary neurons. Statistical analysis was made with the t-student test for unpaired samples (n=4/each experiment).*
